# Advancing Probiotic Delivery in Functional Yogurt: Encapsulation in Prebiotic-Based Matrices

**DOI:** 10.3390/foods14081423

**Published:** 2025-04-20

**Authors:** Konstantina Theodora Laina, Christina Drosou, Georgia Frakolaki, Magdalini Krokida

**Affiliations:** School of Chemical Engineering, Zografou Campus, National Technical University of Athens, 9 Heroon Polytechniou St., 15780 Athens, Greece; konstantinalaina@mail.ntua.gr (K.T.L.); geofrak@chemeng.ntua.gr (G.F.); mkrok@chemeng.ntua.gr (M.K.)

**Keywords:** *Lacticaseibacillus rhamnosus* LGG^®^, encapsulation, electrohydrodynamic processing (electrospraying), freeze drying, probiotic yogurt

## Abstract

The aim of this study was to develop a functional yogurt enriched with encapsulated probiotics with viable cell counts exceeding 10^7^ CFU/g while preserving sensory quality, thereby enhancing health benefits and potentially preventing intestinal barrier dysfunction. *Lacticaseibacillus rhamnosus* LGG^®^ was encapsulated in prebiotic-based matrices for enhanced stability, bioavailability, and controlled release under gastrointestinal conditions. Two encapsulation methods were investigated—the innovative electrohydrodynamic processing (electrospraying) method and conventional freeze drying. The encapsulation matrices were composed of inulin and whey protein isolate. Encapsulation efficiency was determined via microbiological analysis, and the encapsulated structures were characterized using scanning electron microscopy. The efficacy of the encapsulated probiotics was further assessed through exposure to gastrointestinal conditions. Electrosprayed LGG^®^ provided the highest survival rates, up to 76%. Storage stability was evaluated at 4 °C for 105 days and after incorporation in commercial yogurt for 60 days. The sensory characteristics of the different yogurt products were also evaluated. The final products presented acceptable sensory features and final viable counts of 1.6–1.8 × 10^7^ CFU/g. The denser structure of electrosprayed LGG^®^ led to even higher protection. The findings highlight the potential of encapsulation—particularly electrospraying—in developing functional foods with improved probiotic delivery systems, paving the way for health-oriented dairy products.

## 1. Introduction

The human gut is rich in microorganisms that play a crucial role in shaping the host’s immune system. Within this intricate ecosystem, probiotic bacteria are a key component of the gut microbiota, offering significant potential to enhance and restore human health. The growing interest in the consumption of probiotics stems from their potential to improve gut health, modulate immune function, and reduce the risk of various diseases, including metabolic disorders and inflammatory conditions [1,2]. However, a significant challenge in probiotic applications is ensuring their survival during storage and gastrointestinal transit. Exposure to acidic environments, bile salts, oxygen, and temperature fluctuations often results in substantial viability loss, reducing their effectiveness [3].

Yogurt serves as an ideal probiotic delivery system due to its high nutrient content and fermentation-derived bioactivities. However, prolonged storage conditions as well as the harsh gastrointestinal conditions contribute to probiotic degradation [4]. Ensuring a viable probiotic concentration of at least 10^6^–10^7^ CFU/g throughout the product’s shelf life requires advanced stabilization strategies. Encapsulation offers a promising solution by embedding probiotic cells within protective matrices that enhance their resistance to environmental stressors while allowing controlled release in the gastrointestinal tract [5]. This study explores encapsulation using prebiotic-based matrices composed of inulin and whey protein isolate, which provide dual benefits: shielding probiotics and serving as substrates for beneficial gut bacteria.

Two encapsulation techniques were investigated: electrohydrodynamic processing (electrospraying) and freeze drying. Electrospraying, an emerging technology, enables the formation of nano- and microstructured carriers with high encapsulation efficiency and uniform morphology, potentially offering superior protection against acidic degradation [6,7,8]. By adjusting electrospraying conditions—such as the solution flow rate, electrical voltage, and the distance between the nozzle and the collection surface—alongside key physicochemical characteristics of the polymer solution, including viscosity, conductivity, and surface tension, a range of particle morphologies can be achieved [9]. One of the principal benefits of electrospraying is its operation at ambient temperature, which helps preserve the chemical integrity, physical attributes, and biological activity of sensitive bioactive compounds during processing [10,11]. Freeze drying, a conventional approach, is widely used for probiotic preservation but may cause structural damage due to freezing-induced stress [5,7,12]. This study aimed to compare these techniques in terms of encapsulation efficiency, probiotic viability under simulated gastrointestinal conditions, and storage stability.

Previous studies have demonstrated the effectiveness of biopolymeric matrices such as whey protein, alginate, and pullulan in probiotic stabilization, yet research on electrospraying for probiotic delivery in dairy products remains limited. For instance, Feng et al. (2023) highlighted the growing application of electrospraying for encapsulating probiotic cells using protein–polysaccharide combinations, noting its mild processing conditions and ability to improve gastrointestinal survival [13]. Similarly, Ma et al. (2024) developed whey-protein-based electrosprayed microcapsules co-loaded with *Lactiplantibacillus plantarum* and epigallocatechin-3-gallate, demonstrating enhanced probiotic viability under thermal and digestive stress [14]. Dima et al. (2023) also showed that modulating electric field polarity during electrospraying significantly improved probiotic retention by localizing cells within the core of maltodextrin-based microcapsules [15]. In contrast, freeze drying remains a widely adopted method for probiotic stabilization due to its scalability and long-standing industry use. For example, Farahmand et al. (2024) directly compared freeze-dried and coaxial electrosprayed formulations in alginate-based hydrogels [16]. Their findings revealed that while both techniques maintained probiotic viability above 10 ^8^ CFU/g under refrigerated storage and simulated digestion, freeze drying resulted in higher viability loss for *Bifidobacterium animalis* compared to electrospraying [16]. Similarly, Amiri et al. (2021) encapsulated *Lactobacillus acidophilus* in whey protein isolate–lactose blends via electrospraying and showed better retention at 4 °C than in unencapsulated or freeze-dried forms [17]. These recent findings emphasize the growing potential of electrospraying as a flexible and protective encapsulation strategy for probiotics in dairy systems.

Most earlier work has centered on strains like *Bifidobacterium* spp. or *Lactobacillus acidophilus*, leaving a notable gap in evaluating *Lacticaseibacillus rhamnosus* (*L. rhamnosus*) LGG^®^ in real food applications. This study addresses this gap by investigating both electrospraying and freeze drying techniques using inulin and whey protein isolate as encapsulation materials for LGG^®^ in functional yogurt. By examining encapsulation efficiency, structural morphology, survival under gastrointestinal simulation, and sensory impact, this study contributes critical insights into optimizing probiotic delivery in fermented dairy systems.

While the encapsulation of probiotics in dairy products has been previously explored, no prior research has specifically investigated *L. rhamnosus* LGG^®^ using both electrospraying and freeze drying within prebiotic-based matrices. Expanding on this objective, the current study offers a direct comparison of these two encapsulation techniques—an innovative electrohydrodynamic method (electrospraying) and the conventional freeze drying approach—using a combination of inulin and whey protein isolate as the encapsulation material. Furthermore, it investigates the impact of each method on probiotic survival under gastrointestinal conditions, during incorporation into yogurt, and throughout prolonged refrigerated storage. By evaluating encapsulation efficiency, structural characteristics, storage stability, and sensory quality, this research provides a comprehensive assessment of how these encapsulation methods influence probiotic viability and functional food development. The findings offer critical insights into optimizing probiotic delivery systems for enhanced health benefits in fermented dairy products.

## 2. Materials and Methods

### 2.1. Materials and Chemicals

Whey protein isolate and inulin were sourced from Hilmar Ingredients (Hilmar, CA, USA) and Sensus (Lawrence Township, NJ, USA), respectively. Other chemicals, such as lithium chloride, sodium chloride, hydrochloric acid, sodium hydroxide, MRS broth, Mueller–Hinton medium, and agar-agar, were obtained from Merck (Darmstadt, Germany). Enzymes including pepsin, pancreatin, and bile salts were purchased from Sigma-Aldrich (Waltham, MA, USA). All experimental procedures utilized double-distilled, deionized water. The probiotic strain *L. rhamnosus* LGG^®^ was kindly provided by Yiotis S.A. The bacterial culture was grown in MRS broth at 37 °C for 16–18 h under static incubation. Following cultivation, cells were collected by centrifugation at 10,000× *g* for 5 min at 4 °C, washed twice with sterile saline solution, and prepared for encapsulation.

### 2.2. Development of Encapsulated Structures

The encapsulation of *L. rhamnosus* LGG^®^ was carried out using two distinct techniques—electrohydrodynamic processing (electrospraying) and freeze drying—under conditions previously identified as optimum for each method.

#### 2.2.1. Preparation of Polymer Solution

For electrospraying, a polymeric solution composed of whey protein isolate and inulin was prepared by dissolving the materials in water under continuous stirring (500 rpm, 4 h) at room temperature. Preliminary tests indicated that the optimal polymeric ratio was 80:10 *w*/*w* (whey protein isolate/inulin), with a total polymer content of 20% *w*/*w*. Complete solubilization was ensured before encapsulation. Similarly, for the freeze drying technique, the prebiotic–protein blend was composed of whey protein isolate and inulin at a 50:50 weight ratio, also at 20% *w*/*w* total solids.

#### 2.2.2. Electrohydrodynamic Processing (Electrospraying)

Encapsulation was performed using a FluidNatek^®^ system (BioInicia S.L., Paterna, Spain). The probiotic-loaded biopolymer solution was placed in a 10 mL syringe connected to a 0.9 mm stainless steel needle, positioned 10 cm from the collector plate. The process was conducted using a solution flow rate of 0.8 mL/h and an applied voltage of 27 kV. These parameters were selected based on their proven capacity to produce uniform microstructures with high probiotic retention [7,10].

#### 2.2.3. Freeze Drying Method

Probiotic-loaded emulsions were frozen at −80 °C (Panasonic MDF-U3386S, Panasonic Healthcare Co., Ltd., Ehime, Japan) and freeze-dried at a pressure of 0.2 mbar for 72 h (BioBase BK-FD10S tabletop freeze dryer, BioBase Biodustry Co., Ltd., Jinan, China). These conditions were regarded as optimum in terms of maintaining probiotic viability and producing structurally stable encapsulates suitable for incorporation into functional yogurt formulations. The dried encapsulates were pulverized and stored at −30 °C until analysis [12].

### 2.3. Characterization of Encapsulated Structures

Microbiological analysis and scanning electron microscopy (SEM) were used to analyze structural and physicochemical properties, providing insights into encapsulation efficiency and probiotic protection mechanisms.

#### 2.3.1. Encapsulation Efficiency (EE%)

Microbiological analysis was conducted to determine the *L. rhamnosus* LGG^®^ count before and after the encapsulation process through plate enumeration on MRS agar, after aerobic incubation at 37 °C for 48 h [5]. Encapsulation efficiency was calculated using the following equation:

(1) $$EY\;\left(\%\right)=\frac{{logN}_{encapsulated}}{{logN}_{initial}},$$

where *logN_encapsulated_* represents the logarithmic CFU count of LGG^®^ in the encapsulated sample, and *logN_initial_* is the logarithmic CFU count initially added to the solution before the encapsulation process.

#### 2.3.2. Scanning Electron Microscopy (SEM)

The structural surface characteristics of the encapsulated probiotic samples were analyzed using a scanning electron microscope (SEM) (Quanta 200 FEI, Hillsboro, OR, USA). Before imaging, the dried samples were sputter-coated with a gold–palladium layer under vacuum using a coating unit (ACE200, Leica Microsystems, Vienna, Austria) at 18 mA for 105 s. The resulting SEM images were then processed and evaluated using ImageJ software (version 1.54p).

#### 2.3.3. Survival in Simulated Gastrointestinal Conditions

To assess the viability of the encapsulated probiotic strains, simulated gastric juice (SGJ) and simulated intestinal juice (SIJ) solutions were prepared following the methodology described by Laina et al. (2024) [7]. More specifically, the SGJ was formulated by dissolving sodium chloride (NaCl, 9 g/L) and pepsin (3 g/L) in deionized water, with the pH adjusted to 1.5 using 1N HCl. The SIJ solution was prepared by adding pancreatin (2 g/L) and bile salts (6 g/L) to a sodium chloride solution (9 g/L), followed by pH adjustment to 6.0 using sterile 0.1 M NaOH. Both SGJ and SIJ solutions were subsequently sterilized through 0.22 μm membrane filtration.

To evaluate the resistance of encapsulated probiotic strains to simulated gastric conditions, 1.0 g of encapsulated probiotic cells was inoculated into 9.0 mL of sterile SGJ solution and incubated at 37 °C under orbital shaking at 50 rpm for 60 and 120 min. Following incubation, the samples were plated on MRS agar to determine viable cell counts. The release rate of encapsulated probiotic strains from their protective matrices was also assessed under simulated colonic conditions, with the number of released probiotic cells quantified using standard enumeration techniques.

Additionally, to examine the survival rate of encapsulated probiotics in simulated intestinal conditions, 1.0 g of encapsulated probiotic cells was transferred into 9.0 mL of SIJ solution and incubated at 37 °C with orbital shaking at 50 rpm for 60 and 120 min. After incubation, viable probiotic counts were determined using plate enumeration methods.

#### 2.3.4. Stability of Encapsulated *L. rhamnosus* LGG^®^ During Storage at 4 °C

The stability of encapsulated *L. rhamnosus* LGG^®^ during storage at 4 °C was assessed in order to ensure its potential for utilization in the development of probiotic yogurt products. *L. rhamnosus* LGG^®^ viability under refrigeration (4 °C) was determined at certain intervals for 105 days using plate counting methods [18].

#### 2.3.5. Stability of Encapsulated *L. rhamnosus* LGG^®^ After Incorporation in Yogurt Products and Storage at 4 °C

To ensure the quality and functional efficacy of probiotic-enriched yogurt products, a comprehensive storage stability study was conducted to evaluate the viability of *L. rhamnosus* LGG^®^ in both encapsulated and non-encapsulated forms [1,18]. The encapsulated probiotic powders developed in this study were incorporated into commercial conventional yogurt formulations at a concentration of 2% *w*/*w*, ensuring that the functional yogurt product retained desirable sensory properties and a uniform texture, along with a probiotic cell count that met the recommended threshold of 10^7^ CFU/g, in accordance with international standards for probiotic-enriched functional foods [19,20,21]. The survival rate of both free and encapsulated probiotic strains was monitored under controlled refrigerated storage conditions (4 °C) for 60 days, with viability assessed through plate enumeration on MRS agar at predetermined intervals [22,23].

#### 2.3.6. Sensory Evaluation

A sensory evaluation was carried out to assess the organoleptic properties of the probiotic-enriched yogurt samples, focusing on appearance, texture, aroma, taste, and overall acceptability. The evaluation involved a panel of 10 trained individuals, including both male and female participants with prior experience in the sensory analysis of dairy products [24]. The yogurt formulations evaluated included three sample types: one containing *L. rhamnosus* LGG^®^ encapsulated via electrospraying, another with the probiotic encapsulated through freeze drying, and a control sample containing non-encapsulated probiotic cells. All samples were provided in coded form and presented in random order to minimize bias. The sensory testing was conducted under standardized environmental conditions in a dedicated sensory laboratory, with controlled lighting and temperature. Panelists assessed each sample using a structured 9-point hedonic scale, where 1 represented “dislike extremely” and 9 indicated “like extremely” [25]. The collected data were statistically analyzed to examine the influence of the encapsulation method on the sensory perception and overall consumer acceptability of the yogurt products.

### 2.4. Statistical Analysis

All data are presented as mean ± standard deviation. Statistical analyses were performed using SPSS Statistics 21, applying Tukey’s test for multiple comparisons. An analysis of variance (ANOVA) was conducted to determine significant differences between groups, with mean comparisons evaluated using Tukey’s post hoc test at a significance level of α = 0.05.

## 3. Results and Discussion

### 3.1. Characterization of Encapsulated L. rhamnosus LGG^®^

#### 3.1.1. Encapsulation Efficiency (EE%)

The encapsulation efficiency (EE%) of *L. rhamnosus* LGG^®^ was assessed by comparing the viable cell count before and after the encapsulation process, as shown in Table 1. Both freeze drying (FD) and electrospraying (ES) demonstrated high encapsulation efficiency, with LGG^®^-FD achieving 90.41 ± 2.48% and LGG^®^-ES 82.67 ± 1.31%.

These results confirm the protective capability of both methods, with freeze drying yielding slightly better cell retention. This is consistent with previous findings by Moayyedi et al. (2018), who observed superior probiotic retention with freeze drying compared to electrospraying, particularly in whey protein–inulin matrices [26]. Their study reported that freeze drying preserved up to 10.97 log CFU/g of *L. rhamnosus*, with significant declines observed in electrosprayed samples due to electrical and shear stresses.

Electrospraying, while slightly less efficient in terms of EE%, still maintained cell viability above the functional threshold and offered the advantage of producing compact and uniform particles, as also noted by Jayaprakash et al. (2023) [27]. Their comparison of freeze drying, spray drying, and electrostatic spray drying showed that electrospraying preserved more LGG cells during processing than traditional spray drying and approached the protection level of freeze drying [27]. Further, encapsulation in protective matrices like whey protein isolate combined with inulin or prebiotics can enhance survival by creating a barrier against processing stresses. Hossain et al. (2021) demonstrated an encapsulation efficiency of over 91% using freeze drying with a cocoa-based matrix, showing the method’s robustness across different carriers [28].

While electrospraying resulted in approximately 18% viability loss, these results are in line with reports from Azizi et al. (2021), who found that electrosprayed microcapsules had slightly reduced EE% compared to spray- and freeze-dried forms, due to exposure to high voltages and rapid dehydration [29]. These results align with previous research, which reported viability reductions ranging from 1.06 to 1.25 log CFU/mL, further reinforcing the potential of electrospraying as an effective probiotic encapsulation technique [30]. These findings highlight that both techniques are effective for probiotic encapsulation, with freeze drying demonstrating a particularly strong balance between protection and bacterial retention under the conditions tested.

#### 3.1.2. Morphological Characterization of FD and ES Encapsulants

**Figure 1:**
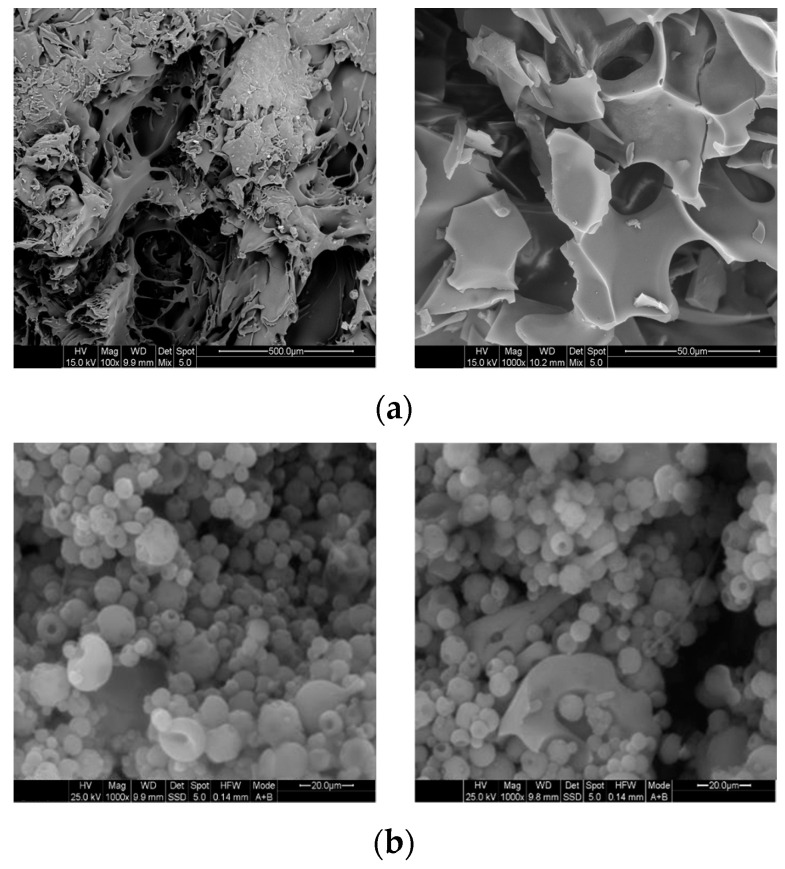
SEM images of encapsulated (**a**) LGG^®^-FD whey protein isolate/inulin (50:50 *w*/*w*) and (**b**) LGG^®^-ES in whey protein isolate/inulin (80:20 *w*/*w*) matrix in 20% *w*/*w* total solid content.

The LGG^®^-FD particles (Figure 1a) exhibited an irregular, porous, and somewhat fragmented surface morphology. This open structure is typical of freeze-dried microcapsules, which may compromise barrier properties and allow greater exposure to environmental stressors, such as oxygen and acidity. In contrast, the LGG^®^-ES microcapsules (Figure 1b) showed more uniform, spherical particles with a smoother, denser outer surface, suggesting enhanced physical integrity and compactness—features that likely contribute to better protective functionality during storage [7,10].

These microstructural observations support the viability data reported below, where LGG^®^-ES retained significantly higher probiotic counts than LGG^®^-FD and free cells during 60-day refrigerated storage in yogurt. The more compact and homogeneous surface of LGG^®^-ES capsules likely limits permeability and provides a better moisture barrier, as also reported by Ying et al. (2010), who observed superior probiotic stability in smoother spray-dried microcapsules compared to porous freeze-dried structures [31]. Similar encapsulation morphology has been documented by Romero-Chapol et al. (2022), where ionic gelation produced spherical, smooth-surfaced capsules that maintained LGG^®^ viability at nearly 100% in yogurt over 30 days [32]. Moreover, Hossain et al. (2021) highlighted that dense structures formed with cocoa powder and Na-alginate enhanced both the thermal and gastrointestinal resilience of LGG^®^ in chocolate formulations [28]. In addition, Qi et al. (2020) found that encapsulated LGG^®^ in alginate hydrogels with chitosan coating had more defined and intact surfaces, which significantly improved storage stability under both ambient and refrigerated conditions [33].

#### 3.1.3. Survival of Encapsulated Probiotic Cells in Simulated Gastric and Intestinal Conditions

The ability of probiotics to withstand acidic conditions and bile salts is a crucial factor in determining their survivability during gastrointestinal transit [26]. To assess the resistance of the encapsulated *L. rhamnosus* LGG^®^ to the harsh environment of the stomach and small intestine, a detailed in vitro gastrointestinal simulation study was conducted. The viability of encapsulated *L. rhamnosus* LGG^®^ (LGG^®^-FD and LGG^®^-ES) was systematically examined under simulated gastric juice (SGJ) and simulated intestinal juice (SIJ) conditions for a duration of 180 min. For comparison, the same test was performed on *L. rhamnosus* LGG^®^ in its free form.

**Figure 2:**
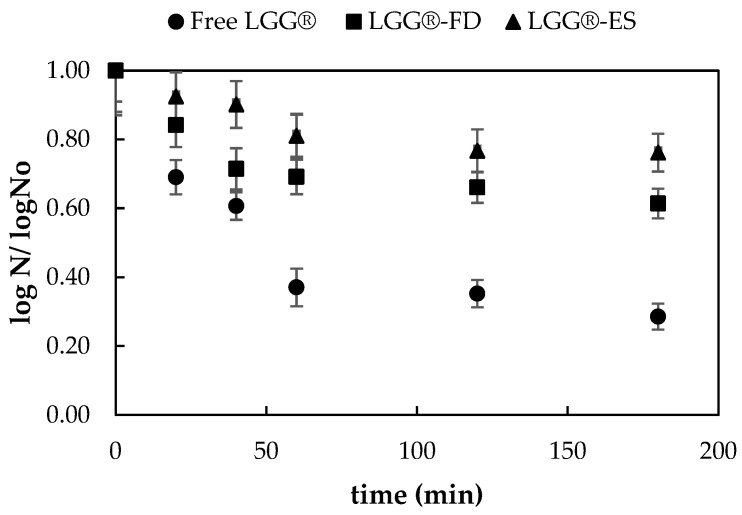
Comparative graph illustrating the viability reduction of *L. rhamnosus* LGG^®^ in free form or encapsulated through freeze drying (LGG^®^-FD) or electrospraying (LGG^®^-ES) following exposure to simulated gastrointestinal conditions.

The results were expressed as the logarithmic reduction in viable cells (log N/log N_0_) before and after exposure to the simulated conditions (Figure 2). Following 60 min in SGJ, free probiotic cells exhibited a significant viability loss of approximately 63%, whereas *L. rhamnosus* LGG^®^-FD and *L. rhamnosus* LGG^®^-ES demonstrated a significantly lower reduction of 30.9% and 18.9%, respectively. The enhanced stability of the encapsulated probiotics may be attributed to the protective properties of the whey protein isolate coating, which likely contributed to shielding the cells from the harsh acidic environment [26,34]. The level of protection provided by encapsulation was influenced by the physicochemical properties of the encapsulating matrix and the encapsulation technique applied. Electrospraying led to even greater resistance of *L. rhamnosus* LGG^®^-ES to the harsh gastric conditions, retaining survival rates at 93%, probably due to the higher surface-area-to-volume ratio that ensures the more uniform and denser encapsulation of *L. rhamnosus* LGG^®^ within the whey protein isolate–inulin matrix, leading to better protection.

A similar trend was observed in simulated intestinal conditions (SIJ). Encapsulated *L. rhamnosus* LGG^®^ maintained higher viability when exposed to 2% bile salt solution at 37 °C for 60 and 120 min. The non-encapsulated *L. rhamnosus* LGG^®^ experienced a viability reduction of approximately 71.4% after 120 min, while the encapsulated *L. rhamnosus* LGG^®^ exhibited a lower viability loss of 23.8–38.6% in total. This suggests that the whey protein isolate–inulin matrix provided substantial protection against bile salt stress. The comparative results highlight the superior performance of encapsulated probiotics, particularly in terms of stability and resistance to gastrointestinal stressors (>61% survival rate), in line with similar studies [35]. The encapsulated *L. rhamnosus* LGG^®^ obtained through electrospraying exhibited enhanced viability, with a survival rate of 76%. This improved stability is likely attributed to the less porous structure of the final particles and the relatively mild processing conditions that helped preserve cell integrity.

The findings of this study confirm that encapsulation effectively protects probiotic cells from gastric acidity, as encapsulated bacteria exhibited significantly higher survival rates than free cells. Additionally, viability decreased progressively over time in the simulated gastrointestinal environment, with non-encapsulated cells showing the most pronounced decline. Thus, encapsulation in a whey protein isolate–inulin matrix appears to be an effective strategy for the development of probiotic yogurt products. It allows for gradual degradation in the stomach, ensuring the maintenance of probiotics’ viability in order to exert their beneficial effects in the intestine [22]. Both methods were proven to be efficient in maintaining the viability of *L. rhamnosus* LGG^®^ cells, with electrospraying providing the greatest protection to the encapsulated cells.

#### 3.1.4. Evaluation of Viability of Encapsulated Probiotic Strains During Storage at 4 °C

**Figure 3:**
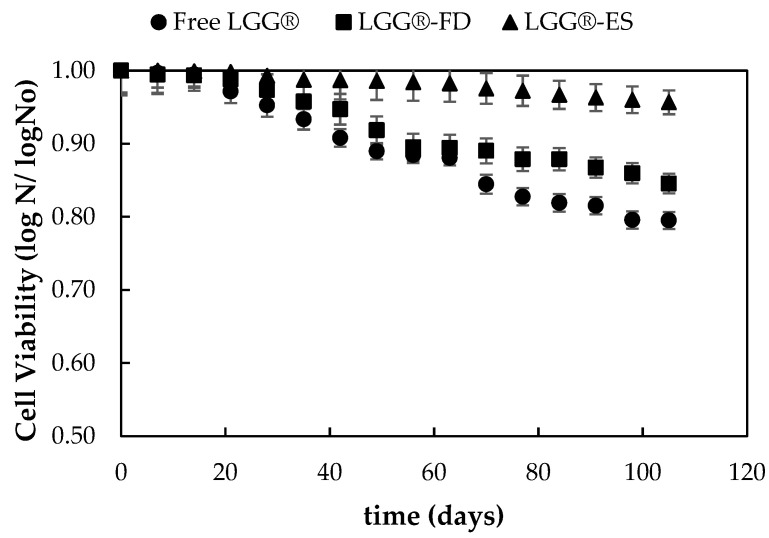
Viability of *L. rhamnosus* LGG^®^ in free form or encapsulated through freeze drying (LGG^®^-FD) or electrospraying (LGG^®^-ES) during storage at 4 °C for 105 days.

The survival rate of free and encapsulated *L. rhamnosus* LGG^®^ was assessed under controlled refrigeration conditions (4 °C) for 105 days. The results of the stability assessment and shelf-life evaluation of the selected encapsulated probiotic strains are presented in Figure 3.

Notably, the encapsulated forms demonstrated significantly higher survival rates than the free cells. LGG^®^ encapsulated via freeze drying (LGG^®^-FD) retained over 85% viability, while electrosprayed LGG^®^ (LGG^®^-ES) maintained more than 88% viability by day 105. These results underscore the protective capabilities of both encapsulation techniques, particularly in preventing oxidative damage and moisture-induced cellular degradation.

The decline in probiotic viability observed in free LGG^®^ samples (falling below 80%) aligns with previous reports highlighting the sensitivity of non-encapsulated probiotics to oxygen exposure, lipid peroxidation, and ambient humidity during refrigerated storage [36,37]. Moayyedi et al. (2018) similarly reported the enhanced survival of *L. rhamnosus* when encapsulated via electrospraying or freeze drying, particularly in whey-protein-based systems [26]. Supporting this, Jayaprakash et al. (2023) found that electrosprayed LGG^®^ exhibited superior viability during storage, attributed to the dense matrix structure and reduced residual moisture [27]. Their work also noted improved outcomes with skim-milk-based carriers due to their oxygen-scavenging and film-forming properties. In a related study, Hossain et al. (2021) reported over 90% viability of freeze-dried LGG^®^ in chocolate matrices after 120 days at 4 °C, demonstrating the method’s suitability for long-term storage [28].

Moisture content and temperature are widely recognized as critical factors influencing probiotic stability during storage [38]. Encapsulation matrices play a crucial role in modulating water activity and oxygen permeability. Freeze drying effectively removes moisture while preserving structural integrity, leading to survival rates consistently above 85%. Electrospraying showed slightly better performance, with viability maintained above 88% at day 105, due to the encapsulant’s ability to buffer against oxidative and hydration-related stress. These findings underscore the fact that the molecular mobility and water content of the encapsulation matrix critically influence long-term probiotic viability, validating their use in functional food applications [1,39,40,41].

#### 3.1.5. Evaluation of Stability and Viability of Encapsulated Probiotic Strains’ Incorporation in Yogurt Products and Storage at 4 °C

**Figure 4:**
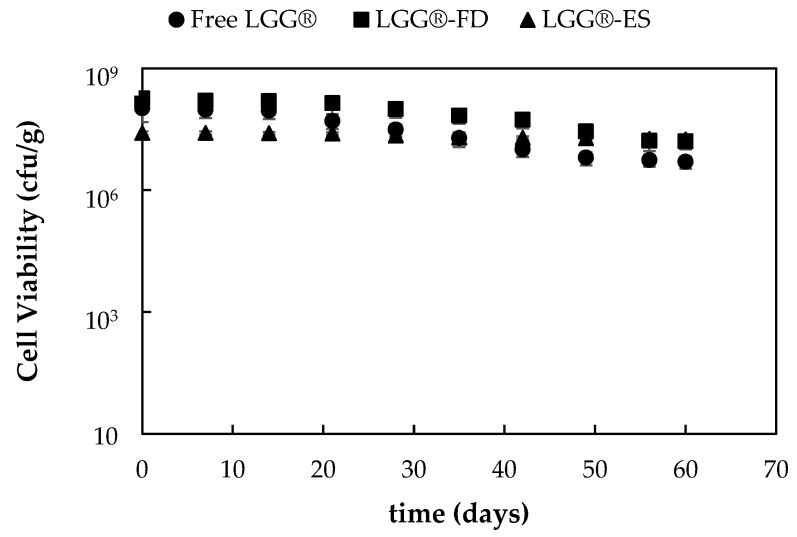
Viability of *L. rhamnosus* LGG^®^ in free form or encapsulated through freeze drying (LGG^®^-FD) or electrospraying (LGG^®^-ES) during incorporation into yogurt formulation and storage at 4 °C for 60 days.

The stability of encapsulated and free *L. rhamnosus* LGG^®^ was further evaluated for 60 days, after incorporation in commercial yogurt formulations. The results of probiotic sensitivity to the acidic environment of yogurt are presented in Figure 4.

Over the 60-day storage period, all samples exhibited a gradual decline in probiotic viability, consistent with the challenges posed by the acidic yogurt matrix. However, encapsulated forms, especially LGG^®^-ES, demonstrated superior stability. The viability loss in the LGG^®^-ES group was significantly lower (approx. 2%) compared to free cells (approx. 12%), with LGG^®^-FD showing intermediate protection. The final viable counts for LGG^®^-ES and LGG^®^-FD were 1.6–1.8 × 10^7^ CFU/g, remaining well above the minimum therapeutic threshold of 10^7^ CFU/g, as recommended by the International Dairy Federation (https://www.researchgate.net/publication/330425199_Inventory_of_microbial_food_cultures_with_safety_demonstration_in_fermented_food_products_Update_of_the_Bulletine_the_IDF_No_455-2012, accessed on 16 April 2025).

These findings corroborate those of previous research, which also observed enhanced survival of encapsulated LGG^®^ in yogurt and fruit matrices during refrigeration [32,42,43]. Specifically, Romero-Chapol et al. (2022) found that encapsulated LGG^®^ maintained 100% viability after 30 days at 4 °C in yogurt, while free cells showed a drop to 92.7%, emphasizing the protective benefits of encapsulation [32]. Similarly, in a study using date yogurt, encapsulated LGG^®^ demonstrated significantly higher survival under refrigeration and gastrointestinal conditions compared to free cells [43]. Oguntoye et al. (2021) also reported that encapsulated LGG^®^ in cassava hydrolysate showed minimal viability loss over 60 days, maintaining >10^7^ CFU/g compared to rapid viability loss in free cells [42].

In terms of encapsulation methods, electrospraying has demonstrated notable advantages over freeze drying. The improved viability in LGG^®^-ES can be attributed to the dense and compact morphology achieved during electrospraying, which provides better protection against oxygen and acidity. This contrasts with the more porous structure of freeze-dried particles, which may allow greater acid penetration, as supported by the comparative morphology findings of Ying et al. (2010), who noted that spray-dried LGG^®^ microcapsules exhibited better survival than freeze-dried variants under humidity stress [31]. Our results also align with Hossain et al. (2021), who demonstrated that encapsulated LGG^®^ in chocolate maintained viability above 10^7^ CFU/g for over 120 days, highlighting the value of optimized encapsulation carriers and techniques [28].

#### 3.1.6. Sensory Evaluation

**Figure 5:**
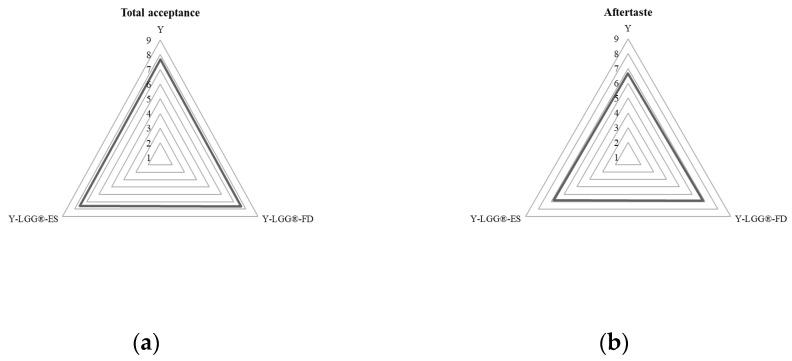
Sensory evaluation scores regarding (**a**) the total acceptance and (**b**) the aftertaste of yogurt formulations containing *L. rhamnosus* LGG^®^ in different forms: plain yogurt without probiotics (Y), yogurt containing LGG^®^ encapsulated via freeze drying (Y-LGG^®^-FD), and yogurt containing LGG^®^ encapsulated via electrospraying (Y-LGG^®^-ES).

The sensory attributes of yogurt formulations containing encapsulated *L. rhamnosus* LGG^®^ were evaluated by a trained panel (n = 10) to assess the impact of encapsulation on consumer-relevant qualities such as appearance, texture, aroma, taste, and overall acceptability [24,25]. As shown in Figure 5, three yogurt variants were assessed: plain yogurt (Y), yogurt with LGG^®^ encapsulated via freeze drying (Y-LGG^®^-FD), and yogurt with LGG^®^ encapsulated via electrospraying (Y-LGG^®^-ES).

The results indicated that all yogurt formulations scored within the acceptable range (scores > 6) for all sensory parameters. However, notable differences were observed between the samples. Yogurt supplemented with LGG^®^ encapsulated via electrospraying (Y-LGG^®^-ES) consistently received the highest scores in terms of texture and appearance, followed closely by Y-LGG^®^-FD, whereas the control sample containing free probiotic cells presented lower ratings, particularly for texture and taste. These findings align with previous studies demonstrating that microencapsulation can mask undesirable sensory effects caused by probiotic additions, such as grittiness or sour off-flavors [39,41,44]. Panelists reported a smoother texture in Y-LGG^®^-ES, potentially attributed to the smaller and more uniform particle morphology achieved through electrospraying, which results in better integration into the yogurt matrix. This observation is supported by prior research indicating that electrosprayed microcapsules exhibit improved dispersion and minimal textural interference in food systems [6,7]. Furthermore, the mild processing conditions of electrospraying, which avoid thermal degradation, may contribute to the preservation of sensory quality and probiotic functionality [13]. Aroma and taste ratings were also slightly higher for encapsulated samples compared to the control, suggesting that encapsulation helped stabilize the probiotic cells and prevented metabolic by-products that may negatively influence flavor. In particular, the electrosprayed yogurt samples exhibited a more neutral and clean taste profile. Similar findings were reported by Adinepour et al. (2022), who noted enhanced flavor stability in dairy products fortified with electrosprayed bioactive compounds [45]. Overall acceptability scores followed a similar trend: Y-LGG^®^-ES > Y-LGG^®^-FD > Y, with statistically significant differences observed between Y-LGG^®^-ES and the non-encapsulated control (*p* < 0.05). These results highlight the sensory benefits of encapsulation in maintaining desirable product characteristics, reinforcing the dual functional role of prebiotic-based encapsulation matrices—not only enhancing probiotic viability, but also improving product quality from a consumer perspective [46]. From an application standpoint, these findings support the feasibility of integrating electrosprayed probiotic encapsulates into yogurt without compromising sensory attributes. The electrospraying technique, in particular, demonstrated superior compatibility with the yogurt matrix, aligning with current industry efforts to develop next-generation functional dairy products that meet both nutritional and sensory expectations [47].

## 4. Conclusions

This study presented a comparative evaluation of two encapsulation techniques—electrohydrodynamic processing (electrospraying) and conventional freeze drying—for the stabilization and delivery of *L. rhamnosus* LGG^®^ in yogurt using a prebiotic-based matrix composed of whey protein isolate and inulin. Both methods were effective in preserving probiotic viability under simulated gastrointestinal conditions, during refrigerated storage, and after incorporation into yogurt. While freeze drying achieved slightly higher encapsulation efficiency (90.41 ± 2.48%), electrospraying produced denser and more uniform microcapsules that provided superior protection under acidic and refrigerated environments. Notably, electrosprayed probiotics maintained viability above the therapeutic threshold (≥10^7^ CFU/g) throughout 60 days of storage and exhibited improved survival under gastrointestinal stress.

Beyond microbial stability, electrosprayed formulations also demonstrated enhanced sensory attributes, receiving significantly higher scores in texture, appearance, and overall acceptability compared to both freeze-dried and non-encapsulated samples. These results underscore electrospraying’s dual advantage in delivering both functional and sensory benefits, making it a promising encapsulation technology for commercial application in probiotic dairy systems. This research provides new insights into the application of electrospraying in real food matrices and represents the first comprehensive study of *L. rhamnosus* LGG^®^ encapsulated via electrospraying in yogurt. Future studies should address scalability, in vivo efficacy, and broader matrix compatibility to support the industrial translation of these findings.

## Figures and Tables

**Table 1:** Encapsulation efficiency (EE%) of *L. rhamnosus* LGG^®^ through FD and ES.

Sample	EE%
LGG^®^-FD	90.41 ± 2.48 ^a^
LGG^®^-ES	82.67 ± 1.31 ^b^

Values labeled with different letters indicate significant differences (*p* < 0.05).

## Data Availability

The original contributions presented in the study are included in the article; further inquiries can be directed to the corresponding author.
